# Integrated Physiological, Transcriptomic and Metabolomic Analyses Provide Insights into the Adaptive Mechanism of *Salix viminalis* Roots in Response to Cadmium Stress

**DOI:** 10.3390/plants15071116

**Published:** 2026-04-05

**Authors:** Jiahui Yin, Jingyi Sun, Mengyao Wan, Baizhou Li, Hang Liu, Rui Yin, Wei Ning

**Affiliations:** 1 Jilin Provincial Key Laboratory of Tree and Grass Genetics and Breeding, College of Forestry and Grassland Science, Jilin Agricultural University, Changchun 130118, China; yinjiahui@jlau.edu.cn (J.Y.); 15543422955@163.com (J.S.); wmyao1429@163.com (M.W.); 13596447373@163.com (B.L.); 2 Key Laboratory of Soil Resource Sustainable Utilization for Jilin Province Commodity Grain Bases, College of Resource and Environmental Science, Jilin Agricultural University, Changchun 130118, China; liuhang0924@163.com; 3 The Zhengzhou Digital Industry Institute, School of Information and Electrical Engineering, Hangzhou City University, Hangzhou 310015, China; yinrui@hzcu.edu.cn

**Keywords:** cadmium, *Salix viminalis*, adaptive mechanism, multi-omics

## Abstract

Cadmium (Cd) is widely dispersed in the environment and has emerged as a major environmental contaminant. Although *Salix viminalis* shows potential for phytoremediation of Cd pollution, the defence mechanism of its roots against heavy metals remains unclear. This study explores the adaptive response of *S. viminalis* roots to Cd stress from physiological, transcriptomic, and metabolomic perspectives. The results suggest that Cd stress exerts inhibitory effects on root growth and development. Compared with the control (Cd-free), the root volume and dry weight of *S. viminalis* exposed to Cd decreased by 26% and 29%, respectively. After exposure to Cd stress for 14 and 21 days, the Cd content in the roots increased by 117-fold and 134-fold, the hydrogen peroxide content increased by 89% and 110%, and the malondialdehyde content increased by 82% and 88%, respectively. This phenomenon can be attributed to the fact that the continuous accumulation of Cd in the roots may have aggravated the degree of lipid peroxidation. A total of 9171 differentially expressed genes (DEGs) and 169 differential metabolites (DIMs) were identified through transcriptomic and metabolomic analyses. Further combined analyses revealed the potential roles of several pathways in the defensive response of *S. viminalis* roots against Cd stress, including plant hormone signal transduction, thiamine metabolism, glycolysis, glycerophospholipid metabolism, and other pathways. Notably, the feedback regulatory effects formed by thiamine metabolism and hormone signal transduction related to auxin, jasmonic acid, and salicylic acid play a crucial role in the early stage when roots are exposed to Cd stress. These effects mobilized osmotic adjustment in roots by enhancing saccharide metabolism and activated the Cd detoxification process by altering lipid metabolism, thereby contributing positively to the defence of willow roots against Cd stress. These findings provide insights into the adaptive mechanism of *S. viminalis* roots in response to Cd and the application of fast-growing woody plants in heavy metal phytoremediation.

## 1. Introduction

With the development of industry and agriculture, the problem of cadmium (Cd) pollution, caused by electroplating, smelting, and the use of low-quality phosphate fertilizers, has posed significant challenges to the healthy development of agriculture [1]. Among the remediation strategies for Cd-contaminated soil, phytoremediation is a highly promising technology with remarkable effectiveness. Trees have a long-term life cycle and relatively high biomass. Over a long period, their tissues can accumulate substantial amounts of heavy metals. *Salix viminalis* is a perennial woody plant characterized by rapid growth, a well-developed root system, large biomass, and strong resistance. Its roots and stems can produce a large quantity of tannins or resins, making it a potential candidate for the remediation of soil composite pollution [2]. However, high concentrations of Cd can inhibit root elongation and reduce the number of lateral roots by interfering with plant physiology and metabolism. This, in turn, decreases the absorption of water and mineral elements in plants, thereby hindering their growth and development [3]. Additionally, excessive absorption of Cd in plants can exacerbate membrane permeability by increasing the levels of superoxide anion radicals (O_2_^·−^) and hydrogen peroxide (H_2_O_2_) through the activation of nicotinamide adenine dinucleotide phosphate (NADPH) oxidase on the cell membrane [4]. Moreover, when Cd ions enter chloroplasts, they disrupt the intracellular reactive oxygen species (ROS) balance by inducing the combination of photosynthetic electrons with oxygen, as they displace metal ions in the photosynthetic apparatus [5]. Consequently, this limits the application of fast-growing woody plants in the field of heavy-metal-contaminated soil remediation.

The roots serve as the primary organ for plants to absorb water and mineral nutrients. They are also the first part of plants to come into contact with toxic metals. The adverse effects of Cd stress on roots are significantly more pronounced than those on other organs [3]. Plants regulate root physiology and metabolism through various hormone-signaling pathways, including those involving auxin (IAA), brassinolide, abscisic acid (ABA), salicylic acid (SA), and jasmonic acid (JA). These hormones regulate the transcription of metal transporter genes and modify the process of root morphogenesis, thereby regulating heavy metal absorption and tolerance. Moreover, through hormone crosstalk, they enhance the antioxidant capacity of plants, thus alleviating oxidative stress induced by heavy metals [6]. For instance, SA improved the osmotic adjustment and antioxidant capacity of *Vigna radiata* by optimizing the water status and increasing the contents of photosynthetic pigments as well as gas exchange parameters. As a result, it reduced the osmotic stress and oxidative damage caused by Cd stress [7]. SA enhanced photosynthetic intensity by increasing the contents of chlorophyll a, b, and carotenoid, thereby mitigating the effects of Cd on *S. viminalis* [8]. And, compared with other willow species, *S. viminalis* demonstrated a greater potential for remediating soil Cd contamination. It did so by increasing the activities of catalase (CAT), ascorbate peroxidase, guaiacol peroxidase [9]. JA alleviated Cd-induced oxidative damage by reducing root Cd absorption and balancing the ROS levels in *Oryza sativa* via increasing the activities of peroxidase (POD), superoxide dismutase (SOD), catalase (CAT), and glutathione reductase [10]. *Arabidopsis thaliana* remodeled its root morphology by upregulating the *auxin resistant 3* and *auxin resistant 1* genes, which were mediated by IAA. Additionally, it increased the total phenolic content to alleviate the toxic effects of Cd on roots [3]. In summary, the regulation of plant hormone signaling is related to the defence response of roots to Cd stress. The root response to adversity directly determines the growth and development status of plants, thereby affecting their heavy metal remediation efficiency.

The advancement of transcriptomic and metabolomic technologies provides an opportunity to unravel the intricate processes underlying plant root responses to heavy metal stress. *Cucurbita moschata* enhanced the activities of SOD and POD in its roots by increasing the levels of metabolites related to carbohydrates, amino acids, unsaturated fatty acids, and IAA, thereby adapting to Cd stress [11]. By upregulating the expression of genes associated with the antioxidant system, as well as heavy metal absorption and accumulation, the SA altered the Cd accumulation, and alleviated oxidative stress, so that it could promote the growth of *Sedum alfredii* under Cd stress [1]. ABA and IAA enhanced CAT activity to mitigate the repression of the *HMA3* and *Nramp5* genes mediated by H_2_O_2_. This enabled them to regulate the adaptive response of *Sedum alfredii* to Cd stress by modifying the Cd accumulation in leaves [6]. *Celosia argentea* adapted to Cd stress by increasing the contents of alanine, proline, and sucrose, and upregulating the expression of leaf metal transporter genes, including *HMA3*, *ABCC15*, and *ATPase4* [12]. In response to Cd stress, *S. viminalis* regulated its roots metabolism, shifting from isoflavonoid production to lignan production. It achieved this by increasing the content of proteins PCBER and DIR5, which are involved in the lignan biosynthetic pathway, and decreasing the content of proteins CHS and CHI, which are involved in the isoflavonoid biosynthetic pathway [13]. However, few studies have explored the molecular mechanisms of *S. viminalis* roots in adaptive response to Cd stress through a multi-omics integrated analysis. Therefore, this study investigated the adaptive response of *S. viminalis* roots to Cd stress from physiological, transcriptomic, and metabolomic perspectives, and explored their intrinsic relevance in root defence against Cd stress.

The findings will contribute to further elucidating the defence mechanisms of fast-growing woody plants in response to Cd stress and providing practical guidance for the application of fast-growing trees in Cd phytoremediation technology.

## 2. Results

### 2.1. Effect of Different Concentrations of Cd on Growth in Salix viminalis

**Figure 1:**
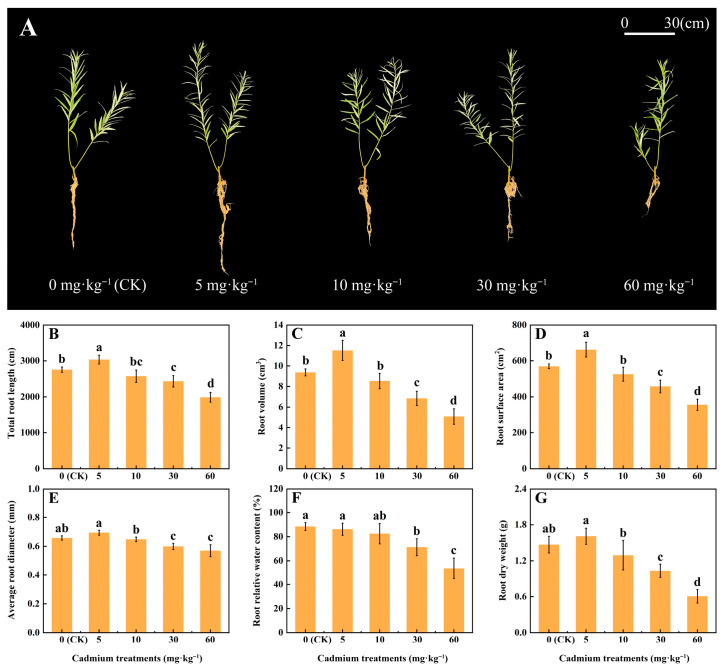
Changes in the root growth parameters and phenotypy of *Salix viminalis* after exposure to Cd stress for 21 days: (**A**) Changes in the phenotypic characteristics of *S. viminalis* under Cd stress; (**B**) Changes in the total root length of *S. viminalis* under Cd stress; (**C**) Changes in the root volume of *S. viminalis* under Cd stress; (**D**) Changes in the root surface area of *S. viminalis* under Cd stress; (**E**) Changes in the average root diameter of *S. viminalis* under Cd stress; (**F**) Changes in the root relative water content (RWC) of *S. viminalis* under Cd stress; (**G**) Changes in the root dry weight of *S. viminalis* under Cd stress. Note: “0 (CK)” indicates Cd-free; different lowercase letters indicate significant difference at 0.05 level among treatments; each value indicates means ± SE (*n* = 4).

After being exposed to different concentrations of Cd for 21 days, the phenotypic characteristics of willow (*S. viminalis*) were showed in Figure 1. As the Cd concentration rose, notable changes occurred in the roots of willows. Compared with the control check (CK, Cd-free), the total root length and volume of the cutting seedlings treated with a 5 mg·kg^−1^ Cd concentration increased. Additionally, the total root length and volume of those treated with 30 and 60 mg·kg^−1^ Cd concentrations decreased. The willow roots were under Cd stress when exposed to a Cd concentration exceeding 30 mg·kg^−1^.

As the Cd concentration increased, the total root length, volume, and surface area exhibited a trend of first increasing and then decreasing. Compared with the CK, the total root length, volume and surface area of the willow treated with a 5 mg·kg^−1^ Cd concentration significantly increased by 10.18%, 22.93%, and 16.38%, respectively (*p* < 0.05). These values significantly decreased by 11.74%, 26.83%, and 19.66% under a 30 mg·kg^−1^ Cd concentration and by 27.77%, 45.64%, and 37.43% under a 60 mg·kg^−1^ Cd concentration, respectively (*p* < 0.05). After Cd treatment, compared with the CK, the average root diameter, relative water content (RWC), and dry weight under a 30 mg·kg^−1^ Cd concentration decreased by 8.97%, 19.49%, and 29.7%, while those under a 60 mg·kg^−1^ Cd concentration decreased by 13.37%, 39.41%, and 58.72%, respectively (*p* < 0.05). It is indicated that high concentrations of Cd inhibited root morphological development and dry matter accumulation in willow and reduced the RWC. Thus, willow roots treated with a 30 mg·kg^−1^ Cd concentration were selected for the subsequent experiments.

### 2.2. Effect of Cd Stress on Physiological Traits of Roots in Salix viminalis

#### 2.2.1. Changes in the Root Osmotic Adjustment Ability of Willow Under Cd Stress

**Figure 2:**
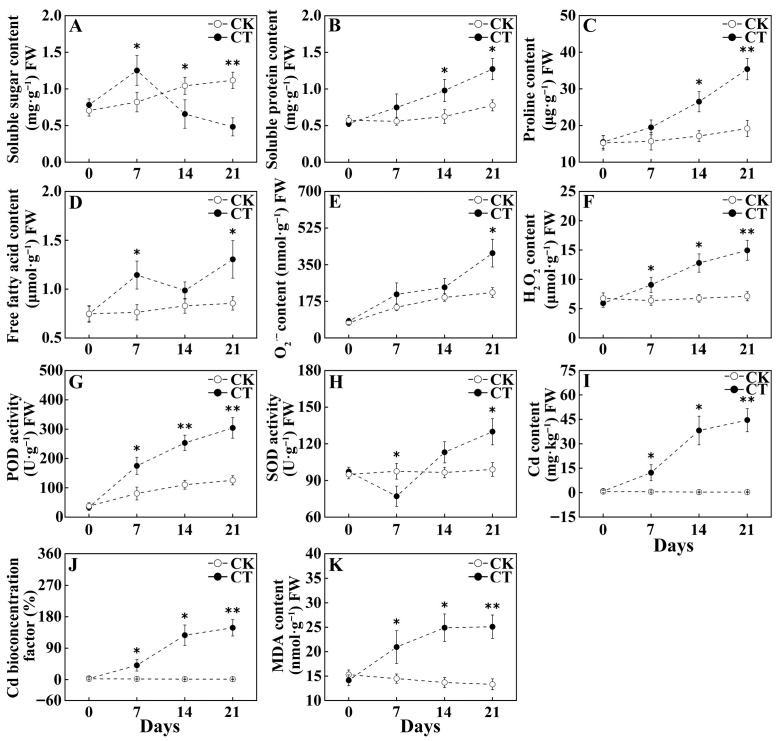
Changes in the root physiological traits of *Salix viminalis* under Cd stress: (**A**) Changes in the content of soluble sugar (SS) in roots under Cd stress; (**B**) Changes in the content of soluble protein (SP) in roots under Cd stress; (**C**) Changes in the content of proline (Pro) in roots under Cd stress; (**D**) Changes in the content of free fatty acid (FFA) in roots under Cd stress; (**E**) Changes in the content of superoxide anion radicals (O_2_^·−^) in roots under Cd stress; (**F**) Changes in the content of hydrogen peroxide (H_2_O_2_) in roots under Cd stress; (**G**) Changes in the activity of peroxidase (POD) in roots under Cd stress; (**H**) Changes in the activity of superoxide dismutase (SOD) in roots under Cd stress; (**I**) Changes in the Cd content in roots under Cd stress; (**J**) Changes in the Cd bioconcentration factor in roots under Cd stress; (**K**) Changes in the content of malondialdehyde (MDA) in roots under Cd stress. Note: “CK” indicates Cd-free, “CT” indicates the willow were exposed to Cd stress, “0” indicates the *S. viminalis* before treatment; “*” indicates a significant difference at 0.05 level between CK and CT, “**” indicates a significant difference at 0.01 level between CK and CT; the values changed over time in each treatment represent repetitions of the same treatment, each value indicates means ± SE (*n* = 4).

The changes in the root physiological traits of *S. viminalis* in response to Cd stress are shown in Figure 2. As time progressed, the soluble sugar (SS) content in the willow roots exhibited a trend of first increasing and then decreasing, whereas the soluble protein (SP) and proline (Pro) contents showed a continuous upward trend (Figure 2A–C). Compared with the CK, the SS content in the willow roots exposed to Cd (CT) significantly increased by 52.56% after 7 days, and then significantly decreased by 36.95% and 56.85% after 14 and 21 days, respectively (*p* < 0.05). The SP and Pro contents in the CT, compared with those in the CK, significantly increased by 56.89% and 54.66% after 14 days of Cd exposure and by 63.92% and 84.5% after 21 days of Cd exposure, respectively (*p* < 0.05). It is indicated that Cd stress initially induced an increase in the SS content in willow roots and subsequently led to an increase in the SP and Pro contents.

#### 2.2.2. Changes in Cd Accumulation in Roots of Willow

The changes in the Cd content in the roots of willow were showed in Figure 2I–J. The Cd content in the roots exposed to Cd increased rapidly after 14 days. The Cd contents in the roots of CT were 22.11-fold, 117.15-fold, and 133.55-fold higher than those in CK after 7, 14, and 21 days of Cd exposure, respectively (Figure 2I). Meanwhile the Cd bioconcentration factor (CBF) in the CT also increased by 22.09-fold, 117-fold, and 133.59-fold, respectively (Figure 2J). It is indicated that the Cd content in the willow roots increased over time.

#### 2.2.3. Changes in Antioxidant Capacity in Roots of Willow Under Cd Stress

The changes in the ROS levels, free fatty acid (FFA) content, lipid peroxidation degree and antioxidant enzyme activity in the roots of willow under Cd stress were showed in Figure 2D–H. Compared with the CK, the O_2_^·−^ content in the roots of CT significantly increased by 86.65% after 21 days of Cd exposure. The H_2_O_2_ contents in the CT also increased significantly by 41.87%, 89.14%, and 109.95% after 7, 14, and 21 days of Cd exposure, respectively (*p* < 0.05). Compared with the CK, the FFA contents in the roots of CT significantly increased by 49.93% and 52.63% after 7 and 21 days of Cd exposure, respectively (*p* < 0.05). The malondialdehyde (MDA) contents in the roots exposed to Cd significantly increased by 44.55%, 81.98%, and 88.41% after 7,14, and 21 days, respectively (Figure 2K) (*p* < 0.05). The POD and SOD activities in the roots exposed to Cd were showed a general trend of rapid increase from day 0 (before treatment) to day 21 (Figure 2G–H). Although the SOD activity in the roots of CT comparing with that in the CK significantly decreased by 20.99% after 7 days of Cd exposure, it still significantly increased by 31.22% after 21 days of Cd exposure (*p* < 0.05). Meanwhile, the POD activity in the roots of CT compared with that in the CK significantly increased by 117.21%, 129.24%, and 152.45% after 7, 14, and 21 days of Cd exposure, respectively (*p* < 0.05). It is indicated that Cd stress initially induced increases in the ROS levels and FFA content in willow roots, inhibited the SOD activity, and elevated the lipid peroxidation degree. Additionally, the Cd stress induced an increase in POD and SOD activities in the willow roots at the later stage of treatment.

#### 2.2.4. Hierarchical Cluster and Pearson Correlation Analysis of Physiological Traits in Willow Roots in Response to Cd Stress

**Figure 3:**
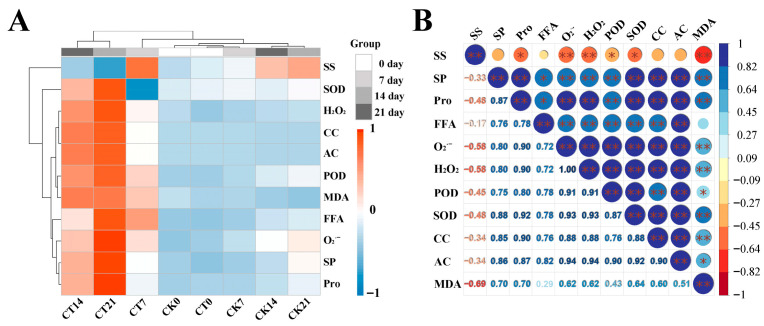
Plots of hierarchical cluster and Pearson correlation in change pattern of physiological traits in *Salix viminalis* roots in response to Cd stress: (**A**) Hierarchical cluster of physiological parameters in roots under Cd stress; (**B**) Pearson correlation analysis of physiological parameters in roots under Cd stress. Note: “CK” indicates Cd-free, “CT” indicates the willow were exposed to Cd stress, “0” indicates the willow before treatment, “7, 14, and 21” indicate the willow after 7, 14, and 21 days of Cd exposure; hierarchical cluster analysis based on the average value means ± SE (*n* = 4); “*” and “**” in Pearson correlation plot indicate significant difference at 0.05 and 0.01 levels, respectively.

To further elucidate the change pattern of physiological traits in *S. viminalis* roots in response to Cd stress, the relationships among samples at different time-points after Cd treatment were analyzed using hierarchical clustering (Figure 3A). In the heat map, the CK groups and the group after 7 days of Cd exposure (CT7) were clustered together on the horizontal axis, while the groups after 14 days (CT14) and 21 days (CT21) were clustered in another group. It appeared that when the willow roots were exposed to Cd stress, short-term changes in their physiological traits were not obvious. As the duration of Cd stress on willow roots increased, the Cd content continuously accumulated in the roots, and the degree of lipid peroxidation in the roots intensified.

The Pearson correlation analysis of physiological traits in willow roots in response to Cd stress was showed in Figure 3B. Throughout the process of willow roots being subjected to Cd stress, the SS contents were negatively correlated with Pro, O_2_^·−^, Cd contents, and CBF (*p* < 0.05). In contrast, the Cd contents were positively correlated with the FFA content (*p* < 0.05), and showed a highly significant positive correlation with SP, Pro, O_2_^·−^, H_2_O_2_ contents, and POD and SOD activities (*p* < 0.01). The MDA contents were positively correlated with O_2_^·−^ and H_2_O_2_ contents. It is indicated that Cd stress inhibited the accumulation of SS in willow roots, induced an increase in ROS levels, and intensified the degree of lipid peroxidation.

### 2.3. Effect of Cd Stress on Root Gene Expression in Salix viminalis

#### 2.3.1. Changes in Root Gene Expression of Willow Under Cd Stress

**Figure 4:**
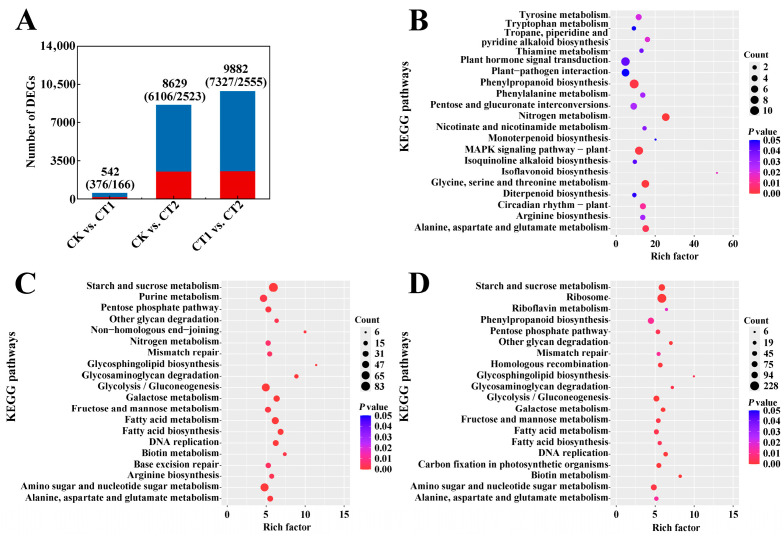
Number distribution and KEGG pathway enrichment plots of DEGs in willow roots under Cd stress: (**A**) Number distribution of DEGs in each comparison groups; (**B**) CK versus (vs.) CT1; (**C**) CK vs. CT2; (**D**) CT1 vs. CT2. Note: each KEGG plot indicated DEG enrichment pathways in the top 20 according to the *p* value (*p* < 0.05); numbers indicate input counts of gene; *p* value indicates temperature range of the gene’s *p* value; “CK” indicates Cd-free, “CT1” indicates the willow were exposed to Cd stress for 12 h, “CT2” indicates the willow were exposed to Cd stress for 36 h; the same below.

The changes in the count distribution of willow root gene expression after Cd exposure were showed in Figure 4A. Compared with the CK, there were 376 downregulated genes (DRGs) and 166 upregulated genes (URGs) after 12 h of Cd exposure (CT1), and 6106 DRGs and 2523 URGs after 36 h of Cd exposure (CT2), respectively. Notably, there were 7327 DRGs and 2555 URGs between the CT1 and CT2 groups, indicating that many genes underwent significant changes within 24 h. Additionally, the willow roots responded to Cd-contaminated environment by regulating these genes, and they might change with an increase in the plant’s Cd absorption concentration.

#### 2.3.2. Changes in Root Gene Enrichment Pathways of Willow Under Cd Stress

The top 20 DEG enrichment pathways across different periods of Cd exposure were identified through Kyoto Encyclopedia of Genes and Genomes (KEGG) enrichment analysis. These pathways were identified basing on the *p* value (*p* < 0.05) and showed in Figure 4B–D. Among these enrichment pathways, the number of pathway terms in the CT2 was far greater than that in the CT1, which could be attributed to the diverse responses of root metabolism to Cd stress at different stages. There were 22 identical KEGG enrichment pathways that appeared in the comparisons of CK versus (vs.) CT1, CK vs. CT2, and CT1 vs. CT2. Compared with CK, more than 7 DEGs were enriched in the “Phenylpropanoid biosynthesis”, “Plant hormone signal transduction”, “MAPK signaling pathway” after 12 h of Cd exposure. After 36 h of Cd exposure, more than 46 DEGs were enriched in the “Starch and sucrose metabolism”, “Plant hormone signal transduction”, “Phenylpropanoid biosynthesis”, “Glycolysis”, “Fatty acid metabolism”, and other pathways. Moreover, between CT1 and CT2, more than 38 DEGs were enriched in the “Starch and sucrose metabolism”, “Galactose metabolism”, “Glycolysis”, “Amino sugar and nucleotide sugar metabolism”, “Fructose and mannose metabolism”, and “Fatty acid metabolism” pathways. It is indicated that willow roots responded to Cd stress by regulating the differential expression of genes associated with hormone signal transduction, saccharide, and fatty acid metabolism at the transcriptional level.

### 2.4. Effect of Cd Stress on Root Metabolites in Salix viminalis

#### 2.4.1. Changes in Root Metabolites of Willow Under Cd Stress

The metabolites of willow roots after Cd exposure were detected through untargeted metabolomic sequencing. The differential metabolites (DIMs) with a *p* value < 0.05 and a variable importance in projection (VIP) score ≥ 1 are shown in Figure S1. There were 36, 133, and 129 DIMs in the comparisons of CK vs. CT1, CK vs. CT2, and CT1 vs. CT2, respectively. Moreover, the number of downregulated DIMs was greater than that of upregulated DIMs in all comparison groups. It is indicated that Cd stress inhibited the synthesis of metabolites in willow roots throughout the entire process. Compared with the CK vs. CT1 group, the number of DIMs in the CK vs. CT2 and CT1 vs. CT2 groups increased significantly.

**Figure 5:**
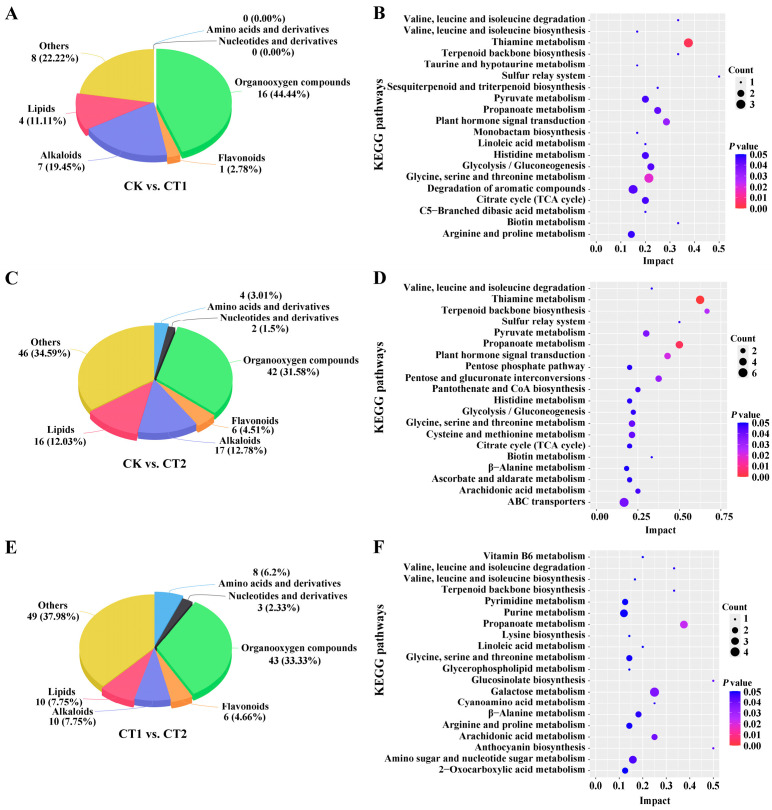
Metabolite classification and KEGG pathway enrichment plots of DIMs in willow roots under Cd stress: (**A**) Metabolite classification of DIMs in CK vs. CT1; (**B**) KEGG plot of CK vs. CT1; (**C**) Metabolite classification of DIMs in CK vs. CT2; (**D**) KEGG plot of CK vs. CT2; (**E**) Metabolite classification of DIMs in CT1 vs. CT2; (**F**) KEGG plot of CT1 vs. CT2. Note: DIMs with *p* < 0.05 and VIP > 1 were classified into seven types; each KEGG plot indicated DIM enrichment pathways in the top 20 according to the *p* value (*p* < 0.05); numbers indicate input counts of metabolite; *p* value indicates temperature range of metabolite *p* value.

To investigate the changes in the types of DIMs in willow roots after exposure to a Cd-contaminated environment, DIMs among the comparison groups were classified into amino acids, nucleotides, organooxygen compounds, flavonoids, alkaloids, lipids, and others (Figure 5A). In the CK vs. CT1 group, the DIM type that accounted for the largest proportion was organooxygen compounds (44.44%). In the CK vs. CT2 group, the top three DIM types were organooxygen compounds (31.58%), alkaloids (12.78%), and lipids (12.03%). The top three types in the CT1 vs. CT2 group were organooxygen compounds (33.33%), alkaloids (7.75%), and lipids (7.75%). Organooxygen and lipid compounds showed significant changes in willow roots under Cd stress. Notably, amino acids and nucleotides only appeared in the roots after 36 h of exposure to the Cd-contaminated environment. It is indicated that the roots of willow might respond to Cd stress by regulating gene expression and amino acid metabolism within a period of 12 to 36 h.

#### 2.4.2. Changes in Root Metabolite Enrichment Pathways of Willow Under Cd Stress

According to the same method, the top 20 DIM enrichment pathways across different periods of Cd exposure, as determined by KEGG analysis basing on the *p* value (*p* < 0.05), were showed in Figure 5B–D. The DIMs mentioned above were mainly enriched in pathways, such as “Thiamine metabolism”, “Glycine, serine and threonine metabolism”, “Plant hormone signal transduction”, “Propanoate metabolism”, “Glycolysis/Gluconeogenesis”, “Citrate cycle (TCA cycle)”, “Pentose and glucuronate interconversions”, “Pyruvate metabolism”, “Galactose metabolism”, “Arachidonic acid metabolism”, and so on. These pathways were related to the DEG enrichment pathways in the roots of willow under Cd stress. Moreover, after 12 h of Cd exposure, more than two DIMs were enriched in the “Thiamine metabolism”, “Glycine, serine and threonine metabolism”, and “Degradation of aromatic compounds” pathways. After 36 h of Cd exposure, more than three DIMs were enriched in the “Thiamine metabolism”, “Propanoate metabolism”, and “ABC transporters” pathways. Meanwhile, four DIMs were enriched in the “Galactose metabolism” pathway between the CT1 and CT2 groups. Some DIMs, such as SA, IAA, thiamine, pyruvate, and thiamine diphosphate (ThPP), simultaneously appeared in the willow roots after 12 and 36 h of Cd exposure. Thus, these compounds may play important roles in the roots of willow in response to Cd.

### 2.5. Correlation Analysis of DEGs and DIMs in Salix viminalis Roots Under Cd Stress

#### 2.5.1. Nine Quadrant Analysis of DEGs and DIMs

To further clarify the model of willow roots in response to Cd stress, the correlation between DEGs and DIMs was analyzed using a nine quadrant plot with a Pearson coefficient ≥0.95, as shown in Figure S2. Compared with the CK vs. CT1 group, the CK vs. CT2 group had more points in quadrants 1, 2, and 4. In contrast, the CT1 vs. CT2 group exhibited several consecutive point matrices in quadrants 1 and 2. This is indicated that the levels of translation or post-translational modification were elevated after willow roots were exposed to Cd stress for 36 h. Moreover, when compared with the CK vs. CT1 group, the CK vs. CT2 group had more points in quadrants 6 and 9, while the CT1 vs. CT2 group showed several consecutive point matrices in quadrants 8 and 9. It is suggested that the transcriptional levels of some genes were increased after willow roots were exposed to Cd stress for 36 h.

#### 2.5.2. KEGG Coinstantaneous Enrichment Analysis of DEGs and DIMs

**Figure 6:**
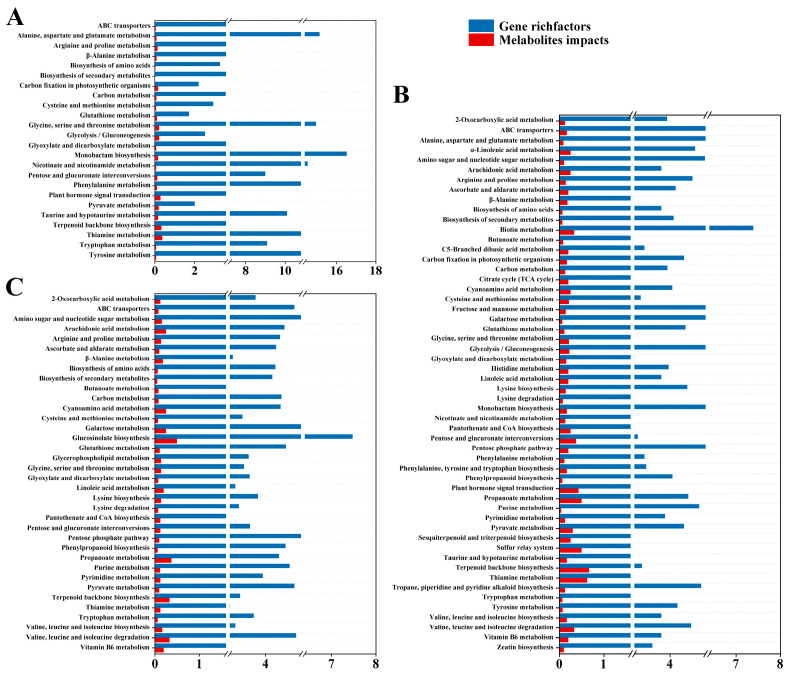
KEGG coinstantaneous enrichment histogram of DEG rich factors and DIM impacts in *Salix viminalis* roots under Cd stress: (**A**) CK vs. CT1; (**B**) CK vs. CT2; (**C**) CT1 vs. CT2. Note: each histogram indicates the common enrichment pathways of DEGs and DIMs among comparison groups.

Based on the KEGG enrichment analysis of DEGs and DIMs in willow roots under Cd stress, the concurrent pathways for each comparison group were showed in Figure 6. There were 24, 54, and 36 concurrent enrichment pathways of DEGs and DIMs in the comparisons of CK vs. CT1, CK vs. CT2, and CT1 vs. CT2, respectively. Moreover, pathways such as “Biosynthesis of amino acids”, “Biosynthesis of secondary metabolites”, “Glyoxylate and dicarboxylate metabolism”, “Pentose and glucuronate interconversions”, “Pyruvate metabolism”, and “Thiamine metabolism”, among others, were observed in all comparison groups. Interestingly, pathways including “α-Linolenic acid metabolism”, “Biotin metabolism”, “Citrate cycle (TCA cycle)”, “Fructose and mannose metabolism”, “Histidine metabolism”, “Phenylalanine, tyrosine and tryptophan biosynthesis”, “Sesquiterpenoid and triterpenoid biosynthesis”, “Tropane, piperidine and pyridine alkaloid biosynthesis”, “Zeatin biosynthesis” were only detected in roots after 36 h of exposure to Cd. It is indicated that the willow roots could adapt to Cd stress by regulating saccharide and lipid metabolism through hormone signal transduction processes.

### 2.6. DEGs and DIMs Associated with Plant Hormone Signal Transduction

**Figure 7:**
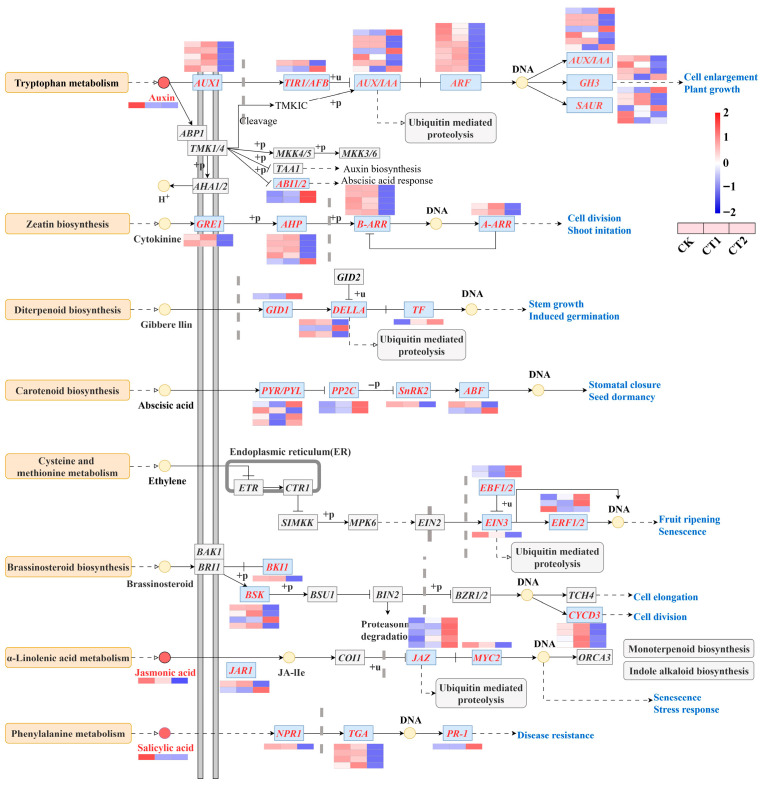
Changes in the root plant hormone signal transduction pathway in *Salix viminalis* under Cd stress. Note: Color key in the upper right corner legend, indicating that temperature range of the gene expression value and metabolite content; each rectangle was divided into three equal parts filling with color, indicating that DEGs and DIMs were regulated in the roots under Cd stress; letters indicate gene symbols that encoded enzymes and symbols refer to abbreviations; the same below.

In the three distinct comparison groups, a total of 91 DEGs were identified (Figure 7). The analysis of DEGs and DIMs associated with plant hormone signal transduction revealed that Cd stress induced multiple hormone signal pathways. The signal pathways of IAA, ABA, JA, and SA were significantly enriched, with many genes exhibiting differential expression. In the IAA signal pathway of roots, the *auxin influx carrier* (*AUX1*), *auxin responsive gene family* (*GH3*), and *auxin response factor* (*ARF*) genes were upregulated after 12 h of exposure to Cd and downregulated after 36 h. Moreover, in the JA signal pathway, the *jasmonic acid-amino synthetase* (*JAR1*) and transcription factor *MYC2* genes were upregulated after 12 h and downregulated after 36 h, while the *jasmonate ZIM domain-containing protein* (*JAZ*) gene was upregulated after 36 h. Additionally, several genes in the ABA and SA signal pathways, such as *pyrabactin resistance* (*PYR*/*PYL*), *protein phosphatase 2C* (*PP2C*), *sucrose non-fermenting 1-related protein kinase 2* (*SnRK2*), *nonexpressor of pathogenesis-related genes 1* (*NPR1*), transcription factor *TGA*, among others, were differentially regulated during these periods. The results of the metabolomics analysis demonstrated that the DIMs related to IAA, JA, and SA were activated.

### 2.7. Thiamine Metabolism in the Roots of Salix viminalis in Response to Cd Stress

**Figure 8:**
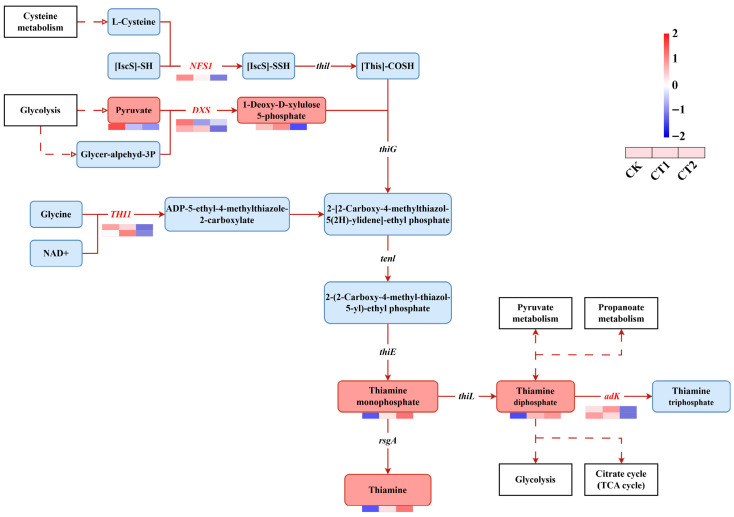
Changes in the root thiamine metabolism in *Salix viminalis* under Cd stress. Note: Symbols refer to abbreviations.

The analysis of DEGs and DIMs enriched in the thiamine metabolism pathway revealed that a total of 7 DEGs and 5 DIMs appeared among the three comparison groups (Figure 8). Compared with the CK, thiamine, thiamine monophosphate, and ThPP continuously accumulated in willow roots during the 12-to-36-h Cd exposure period. Meanwhile, *cysteine desulfurase* (*NFS1*), *1-deoxy-D-xylulose-5-phosphate synthase* (*DXS*), *thiamine thiazole synthase* (*THI1*), and *adenylate kinase* (*adK*) genes showed differential expression in both the CT1 and CT2 groups.

### 2.8. Saccharide Metabolism in the Roots of Salix viminalis in Response to Cd Stress

**Figure 9:**
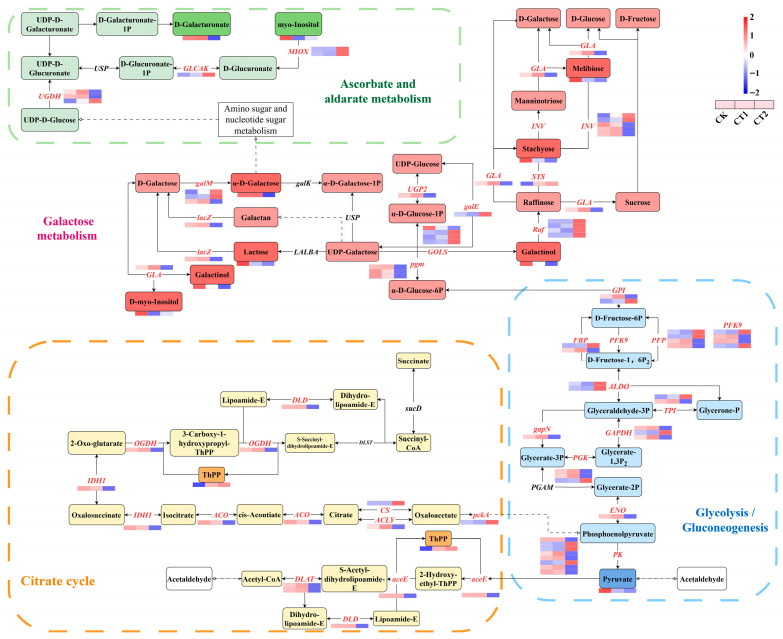
Changes in the root saccharide metabolism in *Salix viminalis* under Cd stress. Note: Symbols refer to abbreviations.

The saccharide metabolism of willow roots in response to Cd stress was showed in Figure 9. A total of 6, 24, 10, and 32 DEGs were identified in “Ascorbate and aldarate metabolism”, “Galactose metabolism”, “Citrate cycle (TCA cycle)”, and “Glycolysis/Gluconeogenesis”, respectively, indicating that these genes were involved in regulating the saccharide metabolism of willow roots in response to Cd. Additionally, a total of 9 DIMs were identified in these pathways. After willow roots were exposed to Cd, compared with the CK, the contents of D-galacturonate, α-D-galactose, and ThPP in CT1 significantly increased, and the content of ThPP in CT2 also increased. Notably, the content of D-myo-inositol in CT2 significantly increased, while the contents of another five DIMs showed a decreasing trend between the CT1 and CT2 groups.

### 2.9. Lipid Metabolism in the Roots of Salix viminalis in Response to Cd Stress

**Figure 10:**
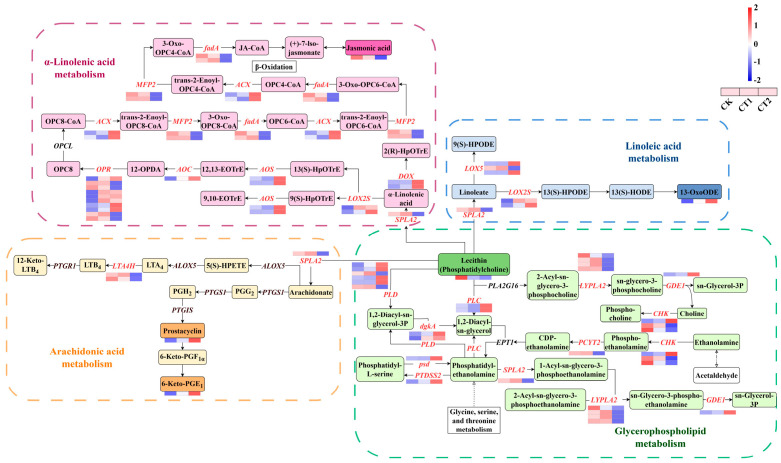
Changes in the root lipid metabolism in *Salix viminalis* under Cd stress. Note: Symbols refer to abbreviations.

The results of transcriptional analysis suggested that the 6 DEGs in the roots of willow after exposure to Cd were enriched in “Linoleic acid metabolism”, 22 DEGs were enriched in “Glycerophospholipid metabolism”, 23 DEGs were enriched in “α-Linolenic acid metabolism”, and 3 DEGs were enriched in “Arachidonic acid metabolism” pathway (Figure 10). Moreover, the metabolic analysis results indicated that as DIMs, 13-OxoODE, phosphatidylcholine, and JA were respectively enriched in “Linoleic acid metabolism”, “Glycerophospholipid metabolism”, and “α-Linolenic acid metabolism” pathways, while prostacyclin and 6-keto-PGE1 were enriched in “Arachidonic acid metabolism” pathway. Compared with the CK, the content of JA in CT1 increased, and the contents of 13-OxoODE, prostacyclin, and 6-keto-PGE1 in CT1 and CT2 also increased.

## 3. Discussion

### 3.1. Salix viminalis Roots Enhanced Osmotic Adjustment and Antioxidant Capacity in Response to Cd Stress

Cd stress exerts inhibitory effects on plant growth and development. For instance, it reduced both shoot height and dry matter accumulation in *Malva parviflora* by inhibiting photosynthesis [14]. Compared with morphogenesis, dry matter accumulation in willow is more sensitive to Cd stress [15]. In this work, the Cd-induced reductions in root total length, volume, surface area, average root diameter, and dry weight collectively indicated that Cd toxicity inhibited willow root growth and development. Under the condition of a 5 mg·kg^−1^ Cd concentration, the increases in root total length, volume, and average root diameter seem to reflect a defensive response of willow roots to cope with Cd stress. It calls for hormetic stimulation of some plant species in response to heavy metals [16].

Generally speaking, the toxic effects of Cd on plants are manifested in two main aspects. On the one hand, Cd^2+^ enters plant guard cells through Ca^2+^ channels, disrupting the balance of cell osmotic potential and inducing osmotic stress [17]. On the other hand, Cd^2+^ enters plant cells via metal transporters, altering the cell membrane structure and disrupting the balance of internal and external ions [14]. Furthermore, Cd^2+^ increases membrane permeability by elevating the contents of O_2_^·−^ and H_2_O_2_ through activating NADPH oxidase in the cell membrane, and leads to an increase in the plant MDA content [4]. The MDA content can serve as an indicator to measure the degree of lipid peroxidation in plant cells under Cd stress [18]. In this work, when treated with a 30 mg·kg^−1^ Cd concentration, the changes in the relative water content of roots indicated that they were dehydrated under high Cd concentration treatment and might be subjected to osmotic stress. As time progressed, the alterations in the contents of O_2_^·−^, H_2_O_2_, and MDA in roots suggested that the increase in ROS levels induced by Cd aggravated the degree of cell lipid peroxidation in roots. Here, the content of FFA in roots exhibited a fluctuating trend over time, indicating that the process of root FFA metabolism in response to Cd stress involved a complex mechanism. This mechanism might be implicated in several processes, such as root cell membrane repair, energy supply, signal transduction, and even the membrane-mediated transport of Cd^2+^ across the cell membrane.

Plants accumulate osmoregulatory substances such as Pro, SS, and SP to cope with the osmotic stress induced by Cd stress [19]. Moreover, SP participates in cellular osmoregulation under Cd stress by being transformed into nitrogen-storage forms [20]. In this study, the changes in the SS content of roots under Cd stress indicated that *S. viminalis* enhanced its osmotic adjustment ability by increasing the SS content during the early stage of Cd stress. Furthermore, the decrease in the SS content in the later stage may be attributed to the excessive accumulation of Cd in plants. Meanwhile, the changes in the contents of SP and Pro in roots suggested that *S. viminalis* responded to Cd-induced osmotic stress by increasing the contents of SP and Pro.

Plants maintain the internal balance of ROS through their own antioxidant enzymes, such as SOD and POD, as well as non-enzymatic antioxidants, so as to cope with Cd stress [21]. In this work, the changes in the activities of SOD and POD indicated that the Cd-induced imbalance in ROS levels surpassed the scavenging capacity of the antioxidant system and also inhibited SOD activity. Furthermore, in the later stage of stress, willow roots might enhance their scavenging ability of O_2_^·−^ by upregulating the expression of SOD-related genes to adapt to Cd. Comparatively, the changes in POD activity suggested that it could effectively balance the H_2_O_2_ levels in root cells under Cd stress. In summary, as the Cd content in the roots continuously accumulated, the willows adapted to the Cd-contaminated environment by enhancing osmotic adjustment, increasing fatty acid content, and boosting antioxidant enzyme activity.

### 3.2. Hormone Signal Transduction in the Roots of Salix viminalis in Response to Cd Stress

The IAA, JA, and SA form a complex regulatory network during the plant’s response to Cd stress. It has been reported that Cd stress induced root structural remodeling by altering the polar transport of IAA in plants. The auxin influx carrier AUX1 is responsible for IAA uptake by cells [22]. After IAA enters the cell, it regulates gene expression changes and signal transduction by targeting the transcriptional repressor protein AUX/IAA and F-box transport inhibitor response 1 (TIR1/AFB) for proteolysis. Then, the auxin response factor (ARF) is in charge of auxin-regulated gene expression of primary response genes. The auxin-responsive gene family *GH3* controls auxin activity through amino acid conjugation, thereby regulating primary root growth [23]. In this study, the changes in *AUX1* and *ARF* indicated that the capacity for IAA uptake and primary response gene expression had changed after the roots were subjected to Cd stress. Meanwhile, the changes in *AUX*/*IAA*, *TIR1*, *GH3* and *SAUR* suggested that willows altered root morphogenesis under Cd stress by regulating the expression of IAA receptors.

The JA signaling pathway can engage in crosstalk with multiple signal transduction pathways, thereby regulating several aspects of plant physiological and metabolic processes in response to Cd stress. Under abiotic stress, plants increase their JA content by activating several key enzymes, such as lipoxygenase (LOX2S), allene oxide synthase (AOS), and 12-oxo-phytodienoic acid reductase (OPR) [24,25]. Subsequently, plants inhibit heavy metal ATPase (HMA) transporters by regulating the basic helix-loop-helix transcription factor MYC2. In this study, the changes in *JAZ* and *MYC2* indicated that willows enhanced their self Cd tolerance by inhibiting the expression of *MYC2*. They may also regulate secondary metabolism by activating the COI1-JAZ-MYC2 complex.

The regulatory mechanism of SA-mediated systemic acquired resistance plays a crucial role in plant responses to abiotic stress. The *nonexpressor of pathogenesis-related genes 1* (*NPR1*) is a key regulatory factor in the SA signaling pathway [26]. Moreover, the transcription factor TGA could improve the Cd tolerance of *Arabidopsis thaliana* by regulating H_2_S levels through mediating the expression of *calmodulin-dependent protein kinase II* [27]. In this study, the changes in *NPR1*, *TGA*, and *PR-1* indicated that the regulation of *NPR1* expression induced by Cd stress activated the expression of root *PR-1*. It might also activate the process of Cd detoxicated metabolism involving *SA-induced GST* by regulating the expression of *TGA*. The interactive mechanism among IAA, JA, and SA in plants represents a highly complex process. Under a variety of adverse conditions, the signal transduction between JA and SA demonstrates antagonism [28]. In contrast, during the process of root morphogenesis, the signal transduction between JA and IAA exhibits synergism [29]. Consequently, plant roots can sense and adapt to Cd stress through these hormone-mediated signal transduction networks. In this study, the gene expression and metabolite contents in root cells associated with the IAA, JA, and SA signal transduction pathways were regulated under Cd stress. These changes indicated that gene expression had a synergistic effect on compound metabolism. Additionally, the alterations in root growth and physiology indicated that these signal transduction pathways were involved in the physiological responses to Cd stress and root morphogenesis processes.

### 3.3. Feedback Regulation Between Hormone Signal Transduction and Thiamine Metabolism in Salix viminalis Roots Under Cd Stress

During the process of plant responses to various adverse conditions, thiamine can directly balance the levels of ROS [30]. In the plant hormone signal transduction pathway, thiamine activates the plant stress defence response by inducing the expression of *PR-1* through the SA defence-signal pathway [31]. During the sensing stage of Cd, the upregulation of thiamine synthesis depends on the regulation of ABA on the genes of *thiamine C synthase* (*THIC*) and *thiamine thiazole synthase* (*THI1*). These enzymes are respectively responsible for the biosynthesis of the pyrimidine and thiazole moieties in thiamine [32]. Thus, the regulation between hormone signal transduction and thiamine metabolism constitutes a feedback regulatory process.

In the thiamine metabolism pathway, cysteine desulfurases (NFS1) are responsible for providing the sulfur required for Fe-S cluster assembly in mitochondria [33]. As the rate-limiting enzyme in isoprenoid biosynthesis, 1-deoxy-D-xylulose-5-phosphate synthase (DXS) catalyzes the conversion of pyruvate and glyceraldehyde-3-phosphate into 1-deoxy-D-xylulose-5-phosphate [34]. Adenylate kinase (adK), a nucleoside monophosphate kinase (NMPK), is responsible for catalyzing a reversible reaction that interconverts ThPP and adenosine diphosphate into thiamine triphosphate and adenosine monophosphate [35]. Here, the changes in the expression levels of *NFS1*, *DXS*, *THI1* and *adK* in roots exposed to Cd stress indicated that Cd induced the upregulation of thiamine synthesis in roots. Meanwhile, the changes in the contents of thiamine, thiamine monophosphate (TMP), and ThPP suggested that roots adapted to the Cd-contaminated environment by increasing thiamine contents. Although no change was detected in the ABA content, the expression of receptor and inhibitor genes, such as *pyrabactin resistance* (*PYR*/*PYL*), *protein phosphatase 2C* (*PP2C*), *sucrose non-fermenting 1-related protein kinase 2* (*SnRK2*) and *ABA response element binding factor* (*ABF*), was upregulated in the ABA signal pathway [36]. This suggested that roots might upregulate thiamine synthesis via the ABA signal pathway to cope with Cd stress. Moreover, based on the changes in the contents of thiamine, TMP, and ThPP, as well as in the IAA, JA, and SA signal pathways, we propose a conceptual model suggesting that there is a feedback regulation between hormone signal transduction and thiamine metabolism in roots under Cd stress.

### 3.4. Salix viminalis Roots Improved Metabolism of Saccharides by Increasing Thiamine Contents Under Cd Stress

Thiamine is crucial for the process of plant saccharide metabolism in response to adverse stress. The rate of thiamine biosynthesis directly determines the activity of thiamine-requiring enzymes and, consequently, the rate of carbohydrate oxidation through the TCA cycle and the pentose phosphate pathways [37].

In the TCA cycle, the pyruvate dehydrogenase E1 component (aceE) serves as the first enzyme of the pyruvate dehydrogenase complex (PDC). It catalyzes the conversion of pyruvate, ThPP, and 2-hydroxy-ethyl-ThPP into S-acetyl-dihydrolipoamide-E (SADE) [38]. Additionally, dihydrolipoyllysine-residue acetyltransferase (DLAT), the second enzyme of the PDC, is responsible for catalyzing the conversion of SADE to acetyl-CoA [39]. Moreover, dihydrolipoyl dehydrogenase (DLD), a multifunctional enzyme located in the mitochondria, can catalyze the interconversion between lipoamide-E and dihydrolipoamide-E [40]. Additionally, under the catalysis of 2-oxoglutarate dehydrogenase (OGDH), ThPP binds with 2-oxo-glutarate (2-OG) and converts it into succinyl-CoA [41]. These processes initiate with the conversion of pyruvate to oxaloacetate, which is then gradually converted to 2-OG. Firstly, citrate synthase (CS) and ATP citrate (pro-S)-lyase (ACLY) are responsible for catalyzing the interconversion between oxaloacetate and citrate. Secondly, aconitate hydratase (ACO) and isocitrate dehydrogenase (IDH1) successively catalyze the production of cis-acontiate, isocitrate, and oxalosuccinate from citrate, ultimately leading to the formation of 2-OG [41]. Moreover, as an energy-consuming reaction, phosphoenolpyruvate (PEP) carboxykinase-ATP (pckA) catalyzes the conversion of oxaloacetate to PEP [42]. Here, the changes in ThPP contents and the expression levels of *aceE*, *DLAT*, *DLD*, and *OGDH* indicated that the willow improved the TCA cycle by increasing root ThPP contents to adapt to the Cd-contaminated environment.

Glycolysis is one of the crucial processes in cellular energy metabolism, and its metabolic status directly determines the ability of plants to adapt to adverse environments. Firstly, phosphoglucomutase (pgm) and glucose-6-phosphate isomerase (GPI) catalyze the interconversion among α-D-glucose-1-phosphate (ADGP), α-D-glucose-6-phosphate (ADG6P), and D-fructose-6-phosphate (DF6P), respectively [43]. The conversion between DF6P and D-fructose-1,6-bisphosphate (DF1BP) is catalyzed by fructose-1,6-bisphosphatase (FBP), ATP-dependent (PFK9) and pyrophosphate-dependent (PFP) phosphofructokinase [44]. Secondly, fructose-bisphosphate aldolase (ALDO) catalyzes the reversible conversion of DF1BP into glyceraldehyde-3-phosphate (GCH3P) and glycerone-phosphate (GOP) [45]. Meanwhile, triosephosphate isomerase (TPI) catalyzes the structural transformation between GCH3P and GOP [46]. Additionally, phosphoglycerate kinase (PGK), phosphorylate-dependent (GAPDH) and NADP^+^-dependent (gapN) glyceraldehyde 3-phosphate dehydrogenase catalyze the interconversion among glycerate-3-phosphate, glycerate-1,3-bisphosphate and GCH3P [47]. Finally, the interconversion of PEP and glycerate-2-phosphate is catalyzed by enolase (ENO). Then, pyruvate kinase (PK), which is regulated by multiple genes, can catalyze the conversion of PEP to pyruvate. Both ENO and PK play positive roles in the plant’s response to heavy metal stress [48,49]. In this study, the changes in the expression levels of *pgm*, *GPI*, *FBP*, *PFK9*, and other related genes indicated that the roots adapted to the Cd-contaminated environment by regulating these glycolysis-related genes. Furthermore, the changes in pyruvate content, as well as the expression levels of *PK* and *ENO* indicated that Cd inhibited the process of root glycolysis by decreasing pyruvate content through inhibiting *PK* and *ENO* expression.

There are several pathways that can provide substrates for “Galactose metabolism”. Firstly, the conversion between ADGP and UDP-glucose (UGL) is catalyzed by UTP-glucose-1-phosphate uridylyltransferase (UGP2) [50]. Subsequently, UDP glucose-4-epimerase (galE) converts UGL to UDP galactose (UGA) [51]. Secondly, UGA is successively converted from lactose and D-galactose (DGA) to galactinol, with the conversions being catalyzed by β-galactosidase (lacZ) and galactinol synthase (GOLS), respectively [52]. The synthesis of raffinose starts with galactinol. It is successively converted from raffinose and stachyose to manniontriose (MNT), and these conversions are catalyzed by raffinose synthase (Raf), stachyose synthetase (STS), and β-fructofuranosidase (INV) [53]. Finally, DGA, galactinol, raffinose, MNT, and melibiose are interconverted through the catalytic effect of galactosidase reaction of α-galactosidase (GLA) [54]. Furthermore, aldose-1-epimerase (galM) can catalyze the conversion of DGA to α-D-galactose [55]. In this study, the changes in genes and metabolites related to galactose metabolism indicated that root galactose synthesis was inhibited by Cd stress. Meanwhile, the changes in the expression levels of *Raf*, *STS*, and *INV* suggested that willow roots improved their antioxidant capacity and adapted to the Cd-contaminated environment by upregulating raffinose metabolism.

In the “Ascorbate and aldarate metabolism” pathway, D-glucuronate (DGL) is generated through the catalytic action of myo-inositol oxygenase (MIOX) on myo-Inositol [56]. Subsequently, the conversion of DGL, UDGL, and UDP-D-glucuronate are catalyzed by glucuronokinase (GLCAK) and UDP-glucose dehydrogenase (UGDH). This process supplies substrates for ascorbic acid metabolism [57,58]. In this study, the changes in the content of D-galacturonate, as well as the expression levels of *MIOX*, *GLCAK*, and *UGDH*, indicated that the roots improved ascorbic acid metabolism by upregulating the expression of genes related to ascorbic acid biosynthesis, thereby adapting to the Cd-contaminated environment. Therefore, based on the aforementioned findings, the roots improve the TCA cycle by increasing the content of thiamine for improving saccharide metabolism.

### 3.5. Salix viminalis Roots Improved Metabolism of Lipids by Increasing Thiamine Contents Under Cd Stress

ThPP catalyzes the conversion of pyruvate to acetyl-CoA, which is the first step of de novo fatty acid biosynthesis [59]. Subsequently, through the process of glycolysis, acetyl-CoA is transformed into acetaldehyde (ACEH), and this is followed by the successive synthesis of polyunsaturated fatty acids (PUFAs). These long-chain PUFAs are rich in carboxyl and hydroxyl cation-exchange sites, as well as carbon-carbon covalent bond binding sites. In plants, they play crucial roles in binding Cd^2+^ and regulating the ROS levels [8].

As the hub of fatty acid metabolism, the synthesis of lecithin within the “Glycerophospholipid metabolism” pathway is partially derived from phosphatidylethanolamine (PHLL) and ACEH. After ACEH is converted to ethanolamine, it undergoes a series of catalytic reactions. First, it is catalyzed by choline/ethanolamine kinase (CHK) and ethanolamine-phosphate cytidylyltransferase (PCYT2), and then it is transformed into PHLL via phosphoenthanolamine and CDP-ethanolamine [60,61]. In addition, the interconversion among lecithin, PHLL, 1,2-diacyl-sn-glycerol (DSG), and DSG-3-phosphate (DSG-3P) is catalyzed by phospholipase C (PLC), diacylglycerol kinase (dgkA), and phospholipase D (PLD) [62]. As a mediator between serine and phosphatidyl metabolism, the interconversion between phosphatidyl-L-serine and PHLL is catalyzed by phosphatidylserine decarboxylase (psd) and phosphatidylserine synthase 2 (PTDSS2) [63]. Another branch of PHLL metabolism is the sn-glycerol-3-phosphate (SG3P) pathway, which can feed into the core of the “Glycerolipid metabolism” pathway. PHLL is catalyzed by phospholipase A (SPLA2), lysophospholipase 2 (LYPLA2), and glycerophosphodiester phosphodiesterase (GDE1) to release dissociated fatty acids and is subsequently converted to SG3P [64,65]. Here, the changes in the expression levels of *CHK*, *PCYT2*, *LYPLA2*, and *SPLA2* suggested that the roots might alleviate oxidant stress by improving glycerophospholipid metabolism through upregulating these genes. Furthermore, the changes in the expression of *PLC*, *dgkA*, and *PLD* indicated that the roots might increase DSG-3P accumulation by upregulating these genes to adapt to the Cd-contaminated environment.

Under the catalysis of SPLA2, lecithin is converted into α-linolenic acid (ALA), arachidonate, and linoleate, respectively, and then enters into other fatty acid synthesis pathways. Depending on whether it contains peroxyhydroxyl and cyclopentane groups, ALA is transformed into three different types of compounds in the “α-Linolenic acid metabolism” pathway. Firstly, ALA is converted to 2(R)-HpOTrE (2HTE) by the action of α-dioxygenase (DOX), and then spontaneously undergoes decarboxylation to form heptadecatrienal [66]. The other two branches involve 13(S)-HpOTrE (13HTE) and 9(S)-HpOTrE (9HTE). These are generated when peroxyhydroxyl binds to the sixth or ninth carbon of ALA and is catalyzed by LOX2S. Subsequently, under the dehydrogenative effect of AOS, 13HTE and 9HTE are converted to 12,13-EOTrE (12ETE) and 9,10-EOTrE, respectively. Secondly, the carbon-chain of 12ETE is cyclized to form the primary structure of a cyclopentane ring under the catalysis of allene oxide cyclase (AOC), enabling 12ETE to be converted into 12-oxo-10,15(Z)-phytodienoic acid. Then, it is successively reduced by OPR to 8-[(1S,2S)-3-Oxo-2-[(Z)-pent-2-enyl]cyclopentyl]octanoic acid (OPC8) [25]. After acetyl groups are incorporated into OPC8, the carbon-chain on the acetyl side of OPC8-CoA is sequentially oxidized by acyl-CoA oxidase (ACX) and 3-hydroxyacyl-CoA dehydrogenase (MFP2), and then the carbon-chain is cleaved by acetyl-CoA acyltransferase (fadA) for the synthesis of JA. These processes are well-known as β-oxidation, which plays an essential role in plant fatty acid degradation, development, and the defence response to stress [67,68]. In this work, the changes in JA content and the expression levels of *MFP2* and *fadA* indicated that the roots activate the defence response to Cd stress by improving β-oxidation through the upregulation of these two genes. Additionally, the changes in the expression of *DOX*, *AOS*, *AOC*, and *ACX* suggested that willow roots improved the synthesis of JA by upregulating these genes to adapt to the Cd-contaminated environment.

The structure of the long-chain carbon in “Linoleic acid metabolism” is similar to that of the eighteen carbon atoms in 2HTE. Linoleate is converted to 13(S)-HPODE and 9(S)-HPODE under the catalysis of LOX2S and linoleate 9S-lipoxygenase 5 (LOX5) [69]. Here, the changes in the expression levels of *LOX2S* and *LOX5* indicated that willow roots might regulate their osmotic adjustment ability by driving plastid remodeling to adapt to the Cd-contaminated environment. Additionally, leukotriene A_4_ (LTA_4_) and leukotriene B_4_ (LTB_4_) from “Arachidonic acid metabolism” play an important role in plant defence response. They can be transformed into each other through the hydrolysis catalyzed by leukotriene-A4 hydrolase (LTA4H) [70]. Thus, these PUFAs may help to maintain the fluidity of root cell lipid membranes and alleviate membranal damage caused by Cd.

In this study, the changes in the contents of 13-OxoODE, prostacyclin, 6-keto-PGE1, and the gene expression related to PUFA metabolism indicated that the roots improved the synthesis of PUFAs by upregulating the expression of these genes. Meanwhile, considering the changes in thiamine contents, it may suggest that willow roots improved lipid metabolism by enhancing the TCA cycle through an increase in thiamine contents.

## 4. Materials and Methods

### 4.1. Experimental Materials and Treatment

The same clone of *S. viminalis* cutting seedlings was sourced from the Genetic Resource Nursery of College of Forestry and Grassland Science at Jilin Agricultural University. Annual branches with a diameter of 1.5 ± 0.5 cm were selected as materials for cutting seedlings and then cut into 12 cm segments. Each segment was cultivated in a substrate composed of a mixture of peat and perlite in a volume ratio of 6:1 within a nutrient pot. The peat used is Danish Pindstrup peat soil, with a specific pH of 5.5 and an organic matter content of approximately 68%. After two weeks of cultivation, healthy seedlings were transplanted into plastic pots filled with the same substrate and continuously cultivated for an additional 30 days. Seedlings of similar height were selected for the subsequent experiments.

The experiments were carried out in a sunlight-glass greenhouse. Specifically, the temperature was maintained at 25 ± 5 °C, the relative humidity ranged from 60% to 70%, and the photoperiod was 15 h with an approximate light intensity of 5000 Lx. The seedlings in plastic pots were weighed daily to maintain the water status of the substrate at 70% of field capacity. The irrigation with Cd solution was used as the treatment. The irrigation solution for the treatments was prepared from CdCl_2_ reagent to form a Cd solution. which prepared from was used for treatments. Using distilled water as the control (CK), a pot experiment was set up with five Cd levels, including 0, 5, 10, 30, and 60 mg·kg^−1^ (the mass ratio of Cd to the dry cultivation substrate).

The growth parameters of *S. viminalis* roots were measured to screen for the Cd concentration that could induce stress after the roots were exposed to Cd for 21 days. Based on the experimental results, an irrigation solution with a Cd concentration of 30 mg·kg^−1^ was selected for the subsequent experiment. To investigate the adaptive response of S. viminalis roots to Cd stress in the early stage, the following steps were carried out. After 12 and 36 h of Cd exposure, six root tips were randomly sampled from each plant, pooled into composite samples, and then flash-frozen in liquid nitrogen. The frozen samples were stored at −80 °C for transcriptomic and metabolomic analyses. Fresh root samples were collected to measure the physiological parameters before treatment and after the roots were exposed to Cd for 7, 14, and 21 days.

### 4.2. Measurement of Growth and Physiological Traits

#### 4.2.1. Determination of Growth Parameters

Four roots of *S. viminalis* were randomly selected from each treatment group. The roots were gently washed with distilled water to remove the cultivation substrate. After the surface water on the roots was dried off, the fresh root weights were measured. Then, the samples were scanned using the root scanning image system WinRHIZO (Regent Instument Inc., Quebec, QC,Canada) to calculate the total root length, volume, surface area, and average root diameter. Root relative water content (RWC) was determined by the drying and weighing method. The root tissues were killed by using oven at 105 °C for 30 min. Subsequently, the oven temperature was set to 80 °C and the samples were dried for 24 h until a constant weight was achieved. Finally, the dry weight of the samples was measured to calculate the relative water content.

#### 4.2.2. Physiological Parameters

To measure physiological parameters, four roots were randomly selected from each treatment group. Fresh samples were placed in a centrifuge tube and ground into a homogenate at 4 °C using a tissue homogenizer. The tissue homogenate was then used for the determination of subsequent indicators. The contents of soluble sugar (SS), soluble protein (SP), proline (Pro), free fatty acid (FFA), malondialdehyde (MDA), superoxide anion radicals (O_2_^·−^), and hydrogen peroxide (H_2_O_2_) were measured using assay kits (Solarbio Life Sciences Inc., Beijing, China). The activities of peroxidase (POD) and superoxide dismutase (SOD) were measured using enzyme activity detection kits (Solarbio Life Sciences Inc., Beijing, China). In accordance with the instructions, physiological parameters were measured with an ultraviolet spectrophotometer (Techcomp Inc., Shanghai, China) by employing specific operating techniques and calculation formulas.

The fresh root samples were gently rinsed with a 10 mmol·L^−1^ EDTA-2Na solution for 30 min to remove the surface Cd ions. The root samples were then dried in an oven following the above-mentioned procedures for measuring the Cd content. Then, the dry samples were ground into a homogeneous dry powder using a ball-mill machine (Tissue Lyser II, Retsch Inc., Haan, Germany) and transferred into a digestion container. A mixture of HNO_3_ and H_2_O_2_ at a volume ratio of 3:1 was added to the homogeneous dry powder for digestion. The containers were then placed in a microwave digestion instrument (MDS-10, Xinyi Inc., Shanghai, China) for 2 h. After the enrichment procedure, the Cd concentration of extractive solution was measured using an inductively coupled plasma optical emission spectrometry instrument (ICP-OES-5110, Agilent Inc., Santa Clara, CA, USA). The Cd bioconcentration factor (CBF) was calculated as the ratio of the Cd concentration in the organ to that in the environment [71].

### 4.3. RNA Extraction, Library Construction, and Sequencing

Total RNA was extracted from four willow roots using the TRIzol reagent kit (Invitrogen Inc., Carlsbad, CA, USA) in accordance with the manufacturer’s protocol. RNA quality was evaluated on an Agilent 2100 Bioanalyzer (Agilent Technologies Inc., Palo Alto, CA, USA) and further verified using RNase-free agarose gel electrophoresis. The samples with RNA integrity number (RIN) value ≥ 7 were used for the subsequent tests. After the extraction of total RNA, eukaryotic mRNA was enriched with Oligo(dT) beads. Subsequently, the enriched mRNA was fragmented into short fragments using fragmentation buffer and reverse-transcribed into cDNA with the NEBNext Ultra RNA Library Prep Kit for Illumina (NEB #7530, New England Biolabs, Ipswich, MA, USA). The purified double-stranded cDNA fragments underwent end repair, A-tailing, and were ligated to Illumina sequencing adapters. The ligation reaction was purified using AMPure XP Beads (1.0×). The ligated fragments were then subjected to size selection via agarose gel electrophoresis and amplified by polymerase chain reaction. The resulting cDNA library was sequenced with each sample undergoing 20 million reads by using the Illumina Novaseq6000 platform by Gene Denovo Biotechnology Co. (Guangzhou, China). The read length was approximately 200 bp. To minimize potential batch effects, the libraries were prepared and sequenced within a single sequencing run.

### 4.4. Transcriptome Assembly and Functional Annotation

The reads obtained from the sequencing machines comprise raw reads that contain adapters or low-quality bases, which would affect subsequent assembly and analysis. Thus, to obtain high-quality clean reads, the reads were further filtered using Fastp (version 0.18.0). The remaining clean reads were then employed for standard RNA-seq analysis. The mapped reads of each sample were assembled with StringTie (version 1.3.1) against the reference genome of *Salix spurpurea* available at https://phytozome-next.jgi.doe.gov/info/Spurpurea_v5_1 (accessed on 21 October 2025). For each transcription region, an FPKM (fragment per kilobase of transcript per million mapped reads) value was calculated using RSEM software (version 1.3.1) to quantify its expression abundance and variations.

### 4.5. Differential Expression and Functional Enrichment Analysis

Differential expression analysis of RNAs was conducted using the edgeR package (version 3.12.1) between two different samples. Transcripts with a false discovery rate (FDR) parameter below 0.05 and an absolute fold change ≥ 2 were considered differentially expressed genes (DEGs). Pearson correlation analysis between parallel experiments was carried out using R (version 4.3.1) to assess the repeatability among samples.

Gene Ontology (GO) enrichment analysis identifies all GO terms that are significantly enriched in DEGs compared to the genome background and filters out the DEGs corresponding to specific biological functions. First, all DEGs were mapped to GO terms in the Gene Ontology database (http://www.geneontology.org/ (accessed on 25 October 2025)). The number of genes was calculated for each term, and significantly enriched GO terms in DEGs compared to the genome background were determined by a hypergeometric test. Kyoto Encyclopedia of Genes and Genomes (KEGG) enrichment analysis aids in further understanding the biological functions of genes. Pathway enrichment analysis identified significantly enriched metabolic pathways or signal transduction pathways in DEGs relative to the whole genome background.

### 4.6. Untargeted Metabolomic Sequencing and Analysis

#### 4.6.1. Sample Preparation and Extraction

The root tissues were immediately and rapidly frozen in liquid nitrogen and ground into a fine powder using a mortar and pestle. A mixture of methanol (Thermo Fisher Scientific Inc., Waltham, MA, USA), acetonitrile (Merck KGaA Inc., Darmstadt, Germany), and H_2_O in a volume ratio of 2:2:1 was added to the homogenized tissue solution for metabolite extraction. To monitor the stability and repeatability of instrument analysis, quality control (QC) samples were prepared by pooling equal volumes of each sample and analyzed alongside the other samples. The mixture was centrifuged at 14,000× *g* at 4 °C for 15 min. The supernatant was dried in a vacuum centrifuge.

#### 4.6.2. Liquid Chromatography and Mass Spectrometry

For liquid chromatography-mass spectrometry analysis, the samples were redissolved in a solvent containing of acetonitrile and water in a volume ratio of 1:1. The compounds were detected using an ultra-high-performance liquid chromatography system (1290 Infinity LC, Agilent Technologies Inc., Palo Alto, CA, USA) coupled to a quadrupole time-of-flight mass spectrometer (TripleTOF 6600 Sciex Inc., Shanghai, China). Chromatographic separation was carried out on an ACQUITY UPLC^®^ HSS T3 column (2.1 × 100 mm, 1.8 μm, Waters Inc., Milford, MA, USA) at 25 °C with a flow rate of 0.3 mL·min^−1^. In the automatic mass spectrometry acquisition mode, the instrument was set to scan over the *m*/*z* range of 25–1000 Da, and the accumulation time for the product ion scan was set at 0.05 s per spectrum. The product ion scan was acquired using information-dependent acquisition (IDA) with the high-sensitivity mode selected.

#### 4.6.3. Metabolites Identification and Statistical Analysis

Peak detection, filtering, and alignment were carried out using the XCMS package in R (version 3.12.0). The metabolites were identified by searching through internal and public databases, including HMDB, MassBank, LipidMaps, and MzCloud).

A variable importance in projection (VIP) score derived from the orthogonal partial least squares (OPLS) model was employed to rank the metabolites that best distinguished between the two groups. The threshold of the VIP score was set at 1. Additionally, the *t*-test was also utilized as a univariate analysis method for screening differential metabolites (DIMs). Metabolites with a *p* value < 0.05 and a VIP score ≥ 1 were considered to be DIMs between the two groups. Subsequently, these DIMs were mapped onto KEGG pathways.

### 4.7. Statistical Analysis

The experimental data were statistically analyzed using Microsoft Excel 2024. One-way analysis of variance (ANOVA) was conducted using SPSS software (version 20.0). The results were expressed as the means ± SE (standard error). Principal component analysis (PCA), Pearson correlation analysis, and hierarchical cluster analysis were performed on the experimental parameters using R (version 4.3.1) software. The figures and plots were performed using Origin 2025.

## 5. Conclusions

This work aims to conduct an integrated analysis of physiological, transcriptomic, and metabolomic data to investigate the response mechanism of willow root from sensing to adapting to cadmium (Cd) stress. The results suggested that the continuous accumulation of Cd in roots may be attributed to the exacerbation of root lipid peroxidation degree. Comparatively, during the early stage of Cd treatment, *Salix viminalis* mobilized osmotic adjustment in roots by improving saccharide metabolism and actively activated the Cd detoxification process by altering lipid metabolism to defend against Cd stress. These processes were mediated by the feedback regulation of hormone signal transduction and thiamine metabolism. They regulated willow roots to adapt to Cd stress by altering root morphology and providing substrates for antioxidant enzyme synthesis. This can be accounted for as follows: Firstly, willows changed the IAA absorption capacity of root cells by upregulating *AUX1* and *ARF*, and modified root morphogenesis by regulating *GH3* and *SAUR*. Additionally, willows improved the synthesis of JA in roots by upregulating *DOX*, *AOS*, *AOC*, and *ACX*, and promoted β-oxidation by upregulating *MFP2* and *fadA*. Meanwhile, willows activated the Cd detoxification metabolism in roots by upregulating *NPR1* and *TGA* to activate the expression of *PR-1*. Secondly, willows enhanced thiamine synthesis by upregulating *NFS1*, *DXS*, *THI1*, and *adK*, and increasing thiamine contents to improve the TCA cycle in roots. Moreover, willows improved the antioxidant capacity of roots by upregulating *lacZ*, *GLA*, *UGP2*, *Raf*, and *STS* in galactose metabolism. Finally, willows enhanced the synthesis of polyunsaturated fatty acids by upregulating the expression of genes related to glycerophospholipid, α-linolenic acid, and linoleic acid metabolism, and increasing the contents of thiamine diphosphate, 13-OxoODE, prostacyclin, and 6-keto-PGE1. This work provides insights into the adaptive mechanism of willow roots in response to Cd stress.

## Data Availability

Dataset available on request from the authors. The raw data supporting the conclusions of this article will be made available by the authors on request.

## Supplementary Materials

The following supporting information can be downloaded at: https://www.mdpi.com/article/10.3390/plants15071116/s1, Figure S1: Hierarchical Cluster of DIMs in *Salix viminalis* roots under Cd stress; Figure S2: Correlation nine quadrant diagrams in the association of DEGs and DIMs among comparison groups.

## Abbreviations

The following abbreviations are used in this manuscript:

*AUX1*	*A**uxin influx carrier gene*
*TIR1/AFB*	*F-box transport inhibitor response 1 gene*
*AUX/IAA*	*Transcriptional repressor protein gene*
*ARF*	*Auxin response factor gene*
*GH3*	*Auxin responsive gene family*
*SAUR*	*Small auxin-up RNA*
*YUCCA*	*Catalytic action of indole-3-pyruvate monooxygenase gene*
*ABI1/2*	*Protein phosphatase 2C subfamily protein gene*
*GRE1*	*Cytokinin receptor gene*
*AHP*	*Histidine-containing phosphotransfer peotein gene*
*B-ARR*	*GARP family transcription factor, ARR-B subfamily gene*
*A-ARR*	*Two-component response regulator ARR-A family gene*
*GID1*	*Gibberellin receptor gene*
*DELLA*	*DELLA protein gene*
*TF*	*Phytochrome-interacting factor gene*
*PYR/PYL*	*P**yrabactin resistance gene*
*PP2C*	*Protein phosphatase 2C gene*
*SnRK2*	*Sucrose non-fermenting 1-related protein kinase 2 gene*
*ABF*	*ABA response element binding factor gene*
*EBF1/2*	*EIN3-binding F-box protein gene*
*EIN3*	*Ethylene-insensitive protein 3 gene*
*ERF1/2*	*Ethylene-responsive transcription factor 1 gene*
*BSK*	*BR-signaling kinase gene*
*BKI1*	*BRI1 kinase inhibitor 1 gene*
*CYCD3*	*Cyclin D3 gene*
*JAR1*	*Jasmonic acid-amino synthetase gene*
*JAZ*	*Jasmonate ZIM domain-containing protein gene*
*MYC2*	*Transcription factor MYC2 gene*
*NPR1*	*Nonexpressor of pathogenesis-related genes 1*
*TGA*	*Transcription factor TGA gene*
*PR-1*	*Acidic SA-dependent pathogenic-associated protein gene*
*NFS1*	*Cysteine desulfurase gene*
*DXS*	*1-deoxy-D-xylulose-5-phosphate synthase gene*
*THI1*	*Thiamine thiazole synthase gene*
*adK*	*Adenylate kinase gene*
*MIOX*	*Myo-inositol oxygenase gene*
*GLCAK*	*Glucuronokinase gene*
*UGDH*	*UDP-glucose dehydrogenase gene*
*aceE*	*Pyruvate dehydrogenase E1 component gene*
*DLAT*	*Dihydrolipoyllysine-residue acetyltransferase gene*
*DLD*	*Dihydrolipoyl dehydrogenase gene*
*pckA*	*Phosphoenolpyruvate carboxykinase-ATP gene*
*CS*	*Citrate synthase gene*
*ACLY*	*ATP citrate (pro-S)-lyase gene*
*ACO*	*Aconitate hydratase gene*
*IDH1*	*Isocitrate dehydrogenase gene*
*OGDH*	*2-oxoglutarate dehydrogenase gene*
*pgm*	*Phosphoglucomutase gene*
*GPI*	*Glucose-6-phosphate isomerase gene*
*FBP*	*Fructose-1,6-bisphosphatase gene*
*PFK9*	*ATP-dependent phosphofructokinase gene*
*PFP*	*pyrophosphate-dependent phosphofructokinase gene*
*ALDO*	*Fructose-bisphosphate aldolase gene*
*TPI*	*Triosephosphate isomerase gene*
*GAPDH*	*Phosphorylate-dependent glyceraldehyde 3-phosphate dehydrogenase gene*
*gapN*	*NADP^+^-dependent glyceraldehyde 3-phosphate dehydrogenase catalyze gene*
*PGK*	*Phosphoglycerate kinase gene*
*ENO*	*Enolase gene*
*PK*	*Pyruvate kinase gene*
*UGP2*	*UTP-glucose-1-phosphate uridylyltransferase gene*
*GOLS*	*Galactinol synthase gene*
*galE*	*UDP glucose-4-epimerase gene*
*lacZ*	*β-galactosidase gene*
*GLA*	*α-galactosidase gene*
*galM*	*Aldose-1-epimerase gene*
*Raf*	*Raffinose synthase gene*
*STS*	*Stachyose synthetase gene*
*INV*	*β-fructofuranosidase gene*
*GDE1*	*Glycerophosphodiester phosphodiesterase gene*
*LYPLA2*	*Lysophospholipase 2 gene*
*CHK*	*Choline/ethanolamine kinase gene*
*PCYT2*	*Ethanolamine-phosphate cytidylyltransferase gene*
*PLD*	*Phospholipase D gene*
*PLC*	*Phospholipase C gene*
*dgkA*	*Diacylglycerol kinase gene*
*psd*	*Phosphatidylserine decarboxylase gene*
*PTDSS2*	*Phosphatidylserine synthase 2 gene*
*SPLA2*	*Phospholipase A gene*
*LOX5*	*Linoleate 9S-lipoxygenase 5 gene*
*LOX2S*	*Lipoxygenase gene*
*LTA4H*	*Leukotriene-A4 hydrolase gene*
*DOX*	*α-dioxygenase gene*
*AOS*	*Allene oxide synthase gene*
*AOC*	*Allene oxide cyclase gene*
*OPR*	*12-oxo-phytodienoic acid reductase gene*
*ACX*	*Acyl-CoA oxidase gene*
*MFP2*	*3-hydroxyacyl-CoA dehydrogenase gene*
*fadA*	*Acetyl-CoA acyltransferase gene*
