# Supplementary material for: Integrated Physiological, Transcriptomic and Metabolomic Analyses Provide Insights into the Adaptive Mechanism of Salix viminalis Roots in Response to Cadmium Stress
Source: Plants (Basel). 2026 Apr 5;15(7):1116. doi: 10.3390/plants15071116 (PMC13074550; doi:10.3390/plants15071116)
Supplement: Supplementary file 1 [file plants-15-01116-s001.zip › Figure S1.pdf]

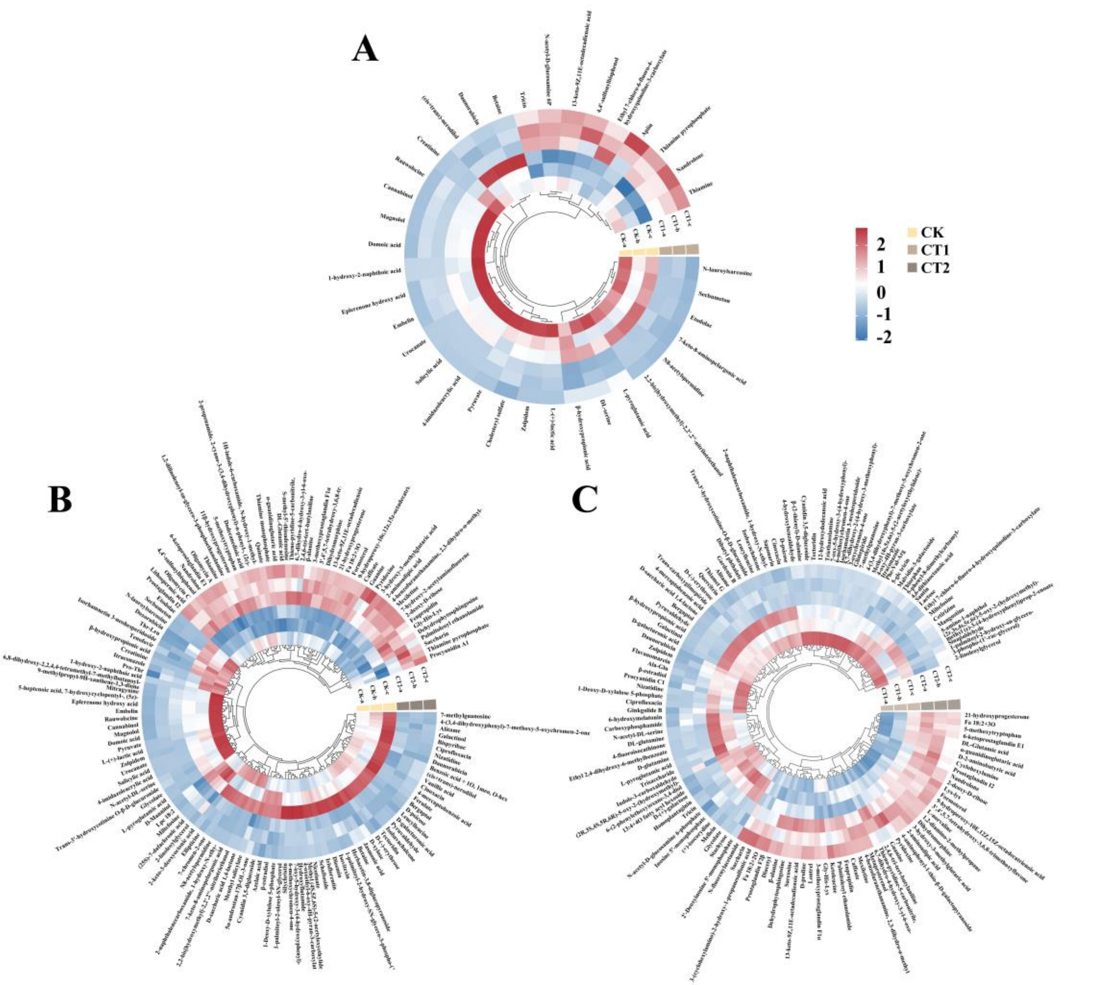


**Figure S1.** Hierarchical Cluster of DIMs in *Salix viminalis* roots under Cd stress. Note: (**A**) CK versus (vs.) CT1; (**B**) CK vs. CT2; (**C**) CT1 vs. CT2. Each plot indicates DIMs among comparison groups according to the $P$ value ($P < 0.05$). "CK" indicates Cd-free, "CT1" indicates the willow were exposed to Cd stress for 12 hours, "CT2" indicates the willow were exposed to Cd stress for 36 hours.
