# Supplementary material for: Integrated Physiological, Transcriptomic and Metabolomic Analyses Provide Insights into the Adaptive Mechanism of Salix viminalis Roots in Response to Cadmium Stress
Source: Plants (Basel). 2026 Apr 5;15(7):1116. doi: 10.3390/plants15071116 (PMC13074550; doi:10.3390/plants15071116)
Supplement: Supplementary file 1 [file plants-15-01116-s001.zip › Figure S2.pdf]

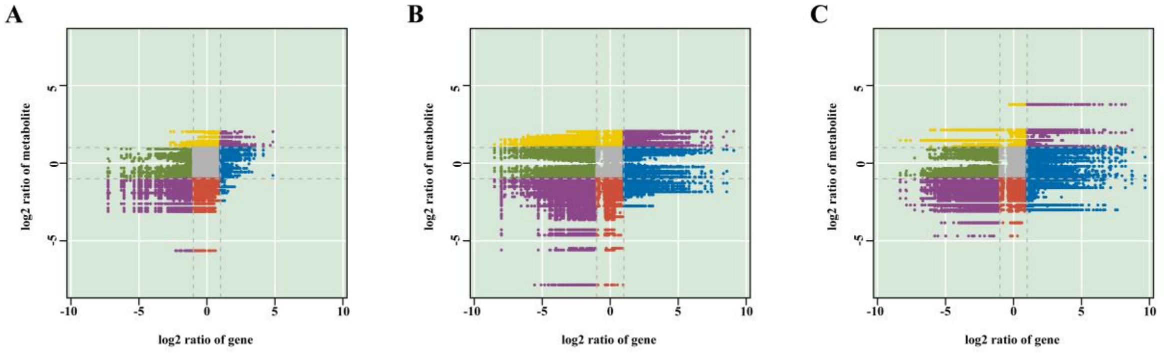

Figure S2. Correlation nine quadrant diagrams in the association of DEGs and DIMs among comparison groups. Note: (**A**) CK versus (vs.) CT1; (**B**) CK vs. CT2; (**C**) CT1 vs. CT2. Each quadrant plots were divided into nine parts, points of each group were filtered by Pearson correlation coefficient ≥ 0.05. In each quadrant plot, the yellow points represent points in quadrants 1 and 2; the purple points represent points in quadrants 3 and 7; the green points represent points in quadrants 4; the grey points represent points in quadrant 5; and the red points represent points in quadrant 8. The points shown in quadrants 1 and 9 indicate DEGs that were negatively associated with DIMs, whereas the other points shown in quadrants 3 and 7 indicate DEGs that were positively associated with DIMs. "CK" indicates Cd-free, "CT1" indicates the willow were exposed to Cd stress for 12 hours, "CT2" indicates the willow were exposed to Cd stress for 36 hours.
